# Case Report: Do not diagnose lung cancer as pneumonia: continue to monitor a case of invasive mucinous adenocarcinoma as it progresses from small to large

**DOI:** 10.3389/fmed.2025.1578874

**Published:** 2025-06-12

**Authors:** Ya-Nan Zhang, Kexin Cao, Yu Yang, Tong Wei, Meng-Jie Li, Jin-Xia Wei, Qiu-Lian Liu

**Affiliations:** ^1^Department of Respiratory and Critical Care Medicine, Renmin Hospital of Qingxian, Cangzhou, China; ^2^Department of Thoracic Surgery, The First Affiliated Hospital of Xinxiang Medical University, Xinxiang, China; ^3^Department of General, General Hospital of Central Theater Command of the People’s Liberation Army, Wuhan, China; ^4^Department of Medical Imaging, Renmin Hospital of Qingxian, Cangzhou, China; ^5^Jiujiang City Key Laboratory of Cell Therapy, Department of Oncology, The First Hospital of Jiujiang City, Jiujiang, China

**Keywords:** invasive mucinous adenocarcinoma, lung cancer, computed tomography imaging, diagnosis, ground-glass opacities lesion, case report

## Abstract

**Background:**

Invasive mucinous adenocarcinoma (IMA) is a rare malignant tumor of the lung, characterized as a distinct subtype of lung adenocarcinoma, with unique histological features and clinical behavior. Because the diagnosis is often delayed due to its imaging characteristics resembling pneumonia, this neoplasm is associated with a poor prognosis.

**Case presentation:**

This case report describes a 69 years-old man who underwent an asymptomatic health examination, during which small ground-glass opacities were found in the periphery of both lungs. There was no significant change in the lesions during the subsequent 2 years follow-up. However, at the third year of follow-up, the lesions had significantly enlarged. A CT-guided puncture biopsy was performed, and the pathological results indicated IMA of the lung. Subsequently, the patient underwent surgical treatment, and the postoperative pathological findings were consistent with those of the biopsy. In this case, the patient believed that he had been following the doctor’s orders for asymptomatic health checks and follow-up reexamination, yet his cancer diagnosis was still significantly delayed. Therefore, the patient demanded that the doctor take medical responsibility for the alleged negligence. After the doctor carefully described the imaging features of lung IMA, the patient ultimately decided to forgo pursuing medical responsibility and expressed satisfaction with the doctor’s diagnosis.

**Conclusion:**

This case illustrates the evolving imaging signs of lung IMA. Medical professionals should avoid diagnosing lung cancer as pneumonia, with the aim of enhancing the accuracy of early diagnosis and assisting in clinical evaluation. Additionally, it serves as a reference for patients to better understand this disease.

## Introduction

Invasive mucinous adenocarcinoma (IMA) is a rare malignant tumor of the lung, characterized by its unique histological and imaging features (1). This subtype of lung adenocarcinoma often presents challenges in diagnosis due to its imaging characteristics, such as airspace opacities and ground-glass opacities (2). It is frequently misdiagnosed as pneumonia due to its pneumonic-type appearance on imaging studies. Early recognition and accurate diagnosis are crucial for improving patient outcomes, as delayed treatment can lead to worse prognoses. The use of advanced imaging techniques, including thin-slice computed tomography (CT) and 18F-FDG PET/CT, has been associated with a high misdiagnosis rate in the differentiation of IMA from pneumonia also (3). Given the rarity of IMA, there is a pressing need for increased awareness among clinicians regarding its potential imaging manifestations and the importance of distinguishing it from other pulmonary conditions. Since there is no previous literature on the persistent evolution of invasive mucinous adenocarcinoma from small lesions, we report such a case here.

## Case report

**FIGURE 1:**
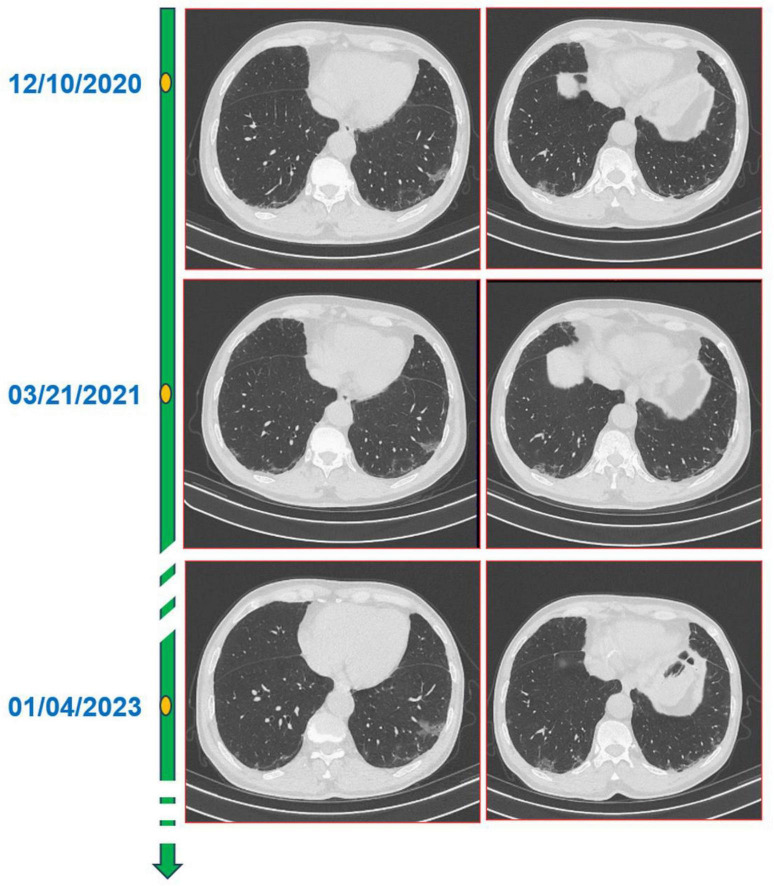
A total of 16-slice computerized tomography images of the chest at initial diagnosis period and in the subsequent 2 years of annual chest computed tomography (CT) re-examination of the patient.

A 69 years-old male patient was hospitalized with lung nodules that had been present for the past 3 years. The patient was found to have small ground-glass opacities in both lungs on chest CT during a screening process for COVID-19 3 years ago (see Figure 1). One lesion in the lower lobe of the left lung was slightly larger, measuring about 0.5 × 0.4 cm, and had an irregular shape. The patient had a temperature of 36.6°C, a respiratory rate of 14 breaths per minute, and a heart rate of 62 beats per minute. The patient and his companion denied that he had asthma, chronic obstructive pulmonary disease, or gastroesophageal reflux disease. He recently denied having a fever, low-grade fever, obvious night sweats, diarrhea, skin-related abnormalities, or edema in his lower extremities. The patient has been smoking for more than 50 years and has not quit, with a daily average of 15 cigarettes. His professional occupation was not related to any special exposure or radiation exposure, and he reported no alcohol consumption, illicit drug use, or recent travel. The patient has no history of pet ownership or unclean sexual contact.

Physical examination showed that the patient had good oral hygiene and no swelling of the tonsils. The patient did not have significantly enlarged lymph nodes, and had a clear respiratory sound in both lungs, and no dry or wet rales heard in either lung field, as well as no pathological murmurs detected during cardiac auscultation. The patient has a history of cerebral infarction for more than 10 years and has been treated with aspirin 100 mg orally once a day. There are currently no special clinical symptoms. The patient denies a history of hypertension, coronary heart disease, diabetes, chronic obstructive pulmonary disease, chronic interstitial pneumonia, and other chronic diseases. The patient also denies allergies to food, drugs, dust, and other substances.

Further examination of the peripheral blood cells counts, liver function, kidney function, electrolytes, myocardial enzymes, blood gas analysis, blood D-dimer, blood glucose, and blood tumor markers all indicated normal results. The patient also underwent tests for erythrocyte sedimentation rate, C-reactive protein, HIV antibody, syphilis antibody, rheumatoid factor, anti-streptococcal hemolysin “O” test, anti-nuclear antibody, anti-double-stranded DNA antibody, and anti-neutrophil cytoplasmic antibody, all of which were normal (see Table 1).

**TABLE 1:** Patient laboratory test results.

Laboratory tests	Normal range	Results
WBC (× 10^9^/L)	4∼10	8.22
C-reactive protein (mg/L)	< 10	6
Neutrophile granulocyte percentage (%)	50∼70	62.1
Eosinophils percentage (%)	0∼5	4.6
Monocytes percentage (%)	3∼8	5.3
Albumin(g/L)	35∼55	38.7
Alanine aminotransferase (U/L)	7∼40	21
Aspartate aminotransferase (U/L)	13∼35	15
Direct bilirubin (μmol/L)	0∼6.8 μmol/L	5.5
Creatinine (μmol/L)	41∼81	54.7
Urea nitrogen (μmol/L)	3.6∼9.5	3.12
Erythrocyte sedimentation rate(mm/h)	0∼20	12
Procalcitonin (ng/mL)	< 0.05	0.02
Interleukin-6 (pg/mL)	0∼40	11
*Mycoplasma pneumoniae* antibody	Negative	Negative
Chlamydia pneumoniae antibody	Negative	Negative
T-SPOT-TB	Negative	Negative
Protein (urinalysis routine)	Negative	Negative
Urobilinogen (urinalysis routine)	Negative	Negative
Occult blood (urinalysis routine)	Negative	Negative
Anti-nRNP/Sm antibody	Negative	Negative
Anti-Sm antibody	Negative	Negative
Anti-SSA antibody	Negative	Negative
Anti-Ro52 antibody	Negative	Negative
Anti-Scl-70 antibody	Negative	Negative
Anti-Jo-1 antibody	Negative	Negative
Anti-CENP B antibody	Negative	Negative
Anti-PCNA antibody	Negative	Negative
Anti-ds DNA antibody	Negative	Negative
Anti- nucleosome antibody	Negative	Negative
Anti-histone antibody	Negative	Negative
Anti-rRNP antibody	Negative	Negative
Anti-AMA M2 antibody	Negative	Negative
Anti-proteinase 3 antibody	Negative	Negative
Anti-myeloperoxidase antibody	Negative	Negative
Anti-neutrophil cytoplasmic antibody-pANCA	Negative	Negative
Anti-neutrophil cytoplasmic antibody-cANCA	Negative	Negative
Serology (colloidal gold-labeled technique)	Negative	Negative

Pulmonary interstitial changes were noted by both respiratory doctors and radiologists. So, the doctor gave the diagnosis of interstitial pulmonary pneumonia. The patient was advised to take oral moxifloxacin 400 mg once daily for 2 weeks. At the same time, the doctor asked the patient to undergo a review 3 months later. At the same time, the doctor asked the patient to undergo a review 3 months later. A chest CT reexamination conducted 3 months later found that the features of both lung lesions were similar to those in the previous health examination images, and the lesions were neither significantly larger nor significantly smaller (see Figure 1).

**FIGURE 2:**
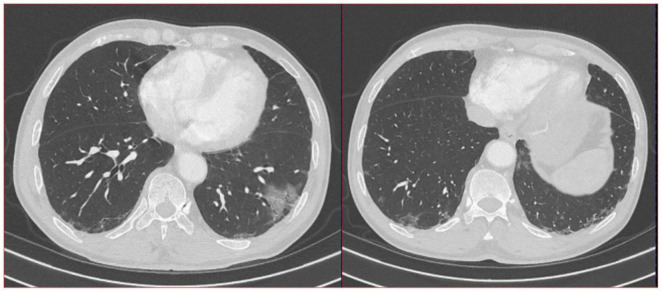
A total of 16-slice computerized tomography images of the chest in the third year (12/27/2023) of re-examination of the patient.

**FIGURE 3:**
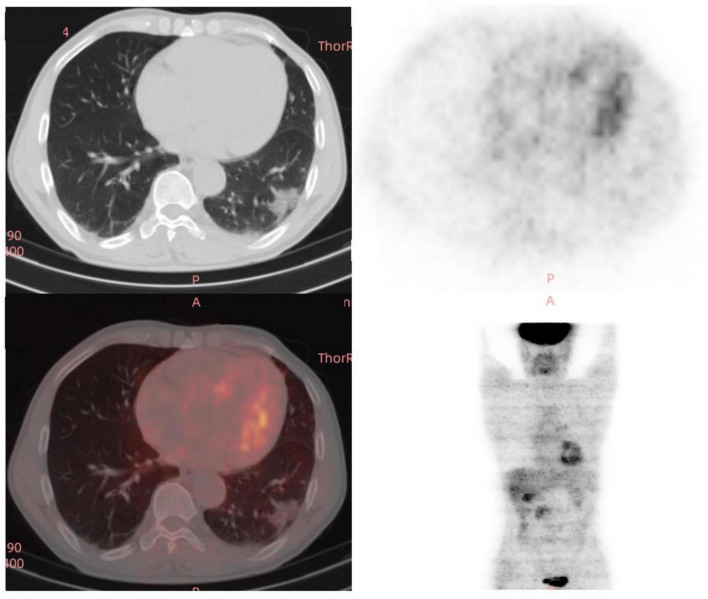
A total of 18 fluoro-D-glucose positron emission tomography/computed tomography (18F-FDG-PET/CT) images of the chest in the third year (12/29/2023) of re-examination of the patient.

The doctors suggested that the patient undergo percutaneous lung biopsy, but the patient refused. So, the patient was recommended a CT reexamination within 1 year. In the subsequent 2 years of annual chest CT reexamination, the imaging changes of both lung lesions were still not significant (see Figure 1). These imaging features did not reveal air bronchogram, and interlobular fissure bulging. However, in the third year of re-examination, chest CT showed that the lesion in the patient’s left lung was significantly larger than before, measuring approximately 3.2 × 3.1 cm. It appears that there are blood vessels running through it, and there is a burr sign (see Figure 2). 18F-FDG PET/CT was performed to identify the nature of lung lesions (see Figure 3). From the images, we can observe that the ground-glass opacities were diffusely distributed in both lungs, especially in the lower lobes of the left lung. The FDG uptake values varied, with a standard uptake value (SUVmax) range of 6.4–24.3, while the SUVmax was 3.7. Which was greater than the SUV value of 2.5 that could be diagnosed as lung cancer. There were no obvious enlarged lymph nodes in the lung hilum or mediastinum.

**FIGURE 4:**
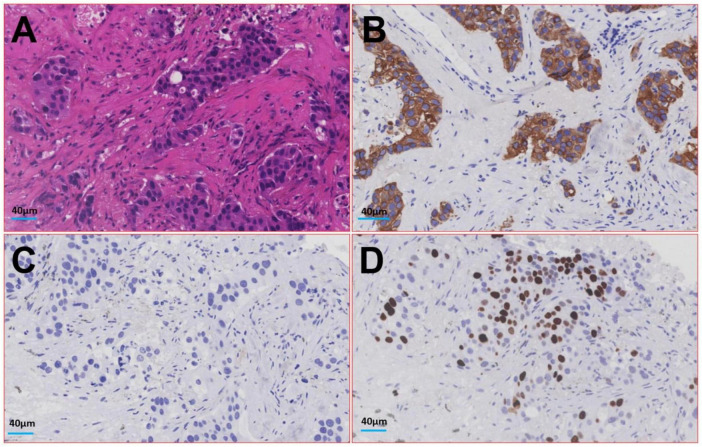
Image of histologic diagnosis from the lesion in the patient’s lower lobe of the left lung. **(A)** Hematoxylin-eosin staining; **(B)** Cytokeratin; **(C)** Thyroid Transcription Factor - 1; **(D)** Ki-67.

Therefore, the radiologist and the respiratory doctor believe that cancer cannot be excluded as the cause of the patient’s lesions. A CT-guided percutaneous lung biopsy was performed, and the lesion was identified microscopically as an IMA of the lung. The tumor cells are in the shape of goblet cells, with abundant mucus in the cytoplasm and nuclei located at the base and perpendicular to the base. Nuclear atypical is not obvious. The surrounding alveolar cavities are filled with mucus. Immunohistochemical examination showed the following results, Cytokeratin (+), Epithelial Membrane Antigen (+), and Thyroid Transcription Factor - 1 (−), with Ki-67 at 10% (see Figure 4). The patient subsequently underwent a left inferior lobectomy, and the postoperative pathological findings were consistent with those of the biopsy tissue.

However, the patient believed that he had been following the doctor’s orders for asymptomatic health checks and follow-up reexamination, yet his cancer diagnosis was still significantly delayed. Therefore, the patient demanded that the doctor take medical responsibility for the alleged negligence. After the doctor carefully described the imaging features of lung IMA, the patient ultimately decided to forgo pursuing medical responsibility and expressed satisfaction with the doctor’s diagnosis and treatment.

## Discussion

Mucinous adenocarcinoma may originate from goblet cells and columnar cells with secretory functions (4). According to the WHO classification of lung adenocarcinoma, carcinoma *in situ* and invasive adenocarcinoma may be accompanied by mucous secretion (5). However, the term “mucinous adenocarcinoma” is specifically used for invasive cases. Mucinous adenocarcinoma may also exhibit various growth patterns, including adherent, acinar, papillary, and micropapillary forms, similar to those seen in common adenocarcinomas (6).

Lung IMA can occur at any age, but it is more common in middle-aged and older individuals. There is no significant difference in the incidence of the disease between men and women, regardless of smoking history (7, 8). Most patients with this disease have no obvious clinical symptoms in the early stage. The main symptoms, when the lesions are significant, include repeated cough and sputum production, primarily white mucus sputum, along with fever. These symptoms are similar to pneumonia but do not respond to anti-inflammatory treatment (9, 10). Laboratory tests may show elevated white blood cell counts and C-reactive protein levels in patients with this disease, though not as significantly as in pneumonia (11, 12).

Imaging findings of lung IMA are critical for diagnosis and management. IMA is a rare subtype of lung adenocarcinoma characterized by distinct radiological features that could aid in differentiating it from other types of lung cancer. Common findings include solid nodules or masses, often with lobulated or spiculated margins. Lung IMA with ground-glass opacities lesion presents challenges in diagnosis (3, 6, 7). Pleural traction is also frequently observed. The imaging findings of it were mainly inferior lobe and dorsal side (gravity distribution characteristics), which easily metastasized along the spread through air spaces (STAS) and led to intrapulmonary dissemination (3). Lesions with unclear boundary of the solid or ground glass shadow, the lesions of the bronchus irregular narrow, rigid, distorted, prone to false cavity sign or vacuole sign (2). In the later stage, the lesions were often mixed in various forms, including consolidation, ground glass, multiple nodules and cystic cavity. Due to the high mucous content in the tumor, the lesion appeared as a lower density shadow than the muscle on CT, which was slightly enhanced on enhanced scan. The median maximum standardized uptake value (SUVmax) of IMA lesions is typically around 3.0, with higher values correlating with advanced disease stages and larger tumor sizes (3). In addition to CT, fluorine-18-fluorodeoxyglucose positron emission tomography (PET)/CT can provide valuable metabolic information (3). The morphologic-metabolic dissociation sign, where a malignant-appearing nodule on CT shows a low SUVmax, has been identified as a potential indicator for distinguishing IMA from invasive non-mucinous adenocarcinomas. Furthermore, thin-slice computed tomography (TSCT) has been shown to effectively classify pulmonary subsolid nodules, helping to differentiate between pre-invasive lesions, minimally invasive adenocarcinomas, and invasive adenocarcinomas (13). The morphologic-metabolic dissociation sign, based on CT and PET/CT, has been evaluated for its efficacy in distinguishing IMA from invasive non-mucinous adenocarcinomas of the lung (3).

Misdiagnosis of lung IMA can lead to significant clinical implications, as this rare subtype of lung cancer often presents with symptoms that mimic other conditions, such as pneumonia. Both of the two kind diseases have flaky, ground-glass shadows on the image features and can even be diagnosed as viral pneumonia if the pneumonia virus is prevalent (14–16). Moreover, in this case, the patient had radiographic findings of lesions in both lungs. In order to obtain a more accurate diagnosis for these lesions, invasive procedures such as thoracoscopic or open lung biopsy, CT-guided percutaneous lung biopsy, and transbronchial lung biopsy are required to obtain lung tissue. According to a case report, both surgical lung biopsy and CT-guided percutaneous lung biopsy had a diagnostic rate of 100%, whereas transbronchial lung biopsy with bronchoalveolar lavage had a rate of 80.9% (14, 17). So, the patient believed that he had been following the doctor’s orders for asymptomatic health checks and follow-up reexamination, yet his cancer diagnosis was still significantly delayed. After we carefully informed and popularized the diagnostic difficulty and current situation of IMA, the patient expressed satisfaction with the doctor’s diagnosis and treatment.

Of course, other technologies, including but not limited to artificial intelligence, microbial genetic testing, and virological antigen detection, all help in the two kind diseases identification (18–20). One of the most common genetic mutations found in IMA is the KRAS mutation, which is present in a significant proportion of cases. This mutation is often accompanied by other genetic alterations, such as those in the STK11 gene, which can further influence the tumor’s behavior and response to treatment (21). The genetic landscape of IMA is further characterized by a low overall mutation burden, which is atypical compared to other lung adenocarcinomas. This low mutation burden is associated with a distinct mutational signature, predominantly linked to endogenous mutational processes rather than external factors like smoking. This suggests that the pathogenesis of IMA may involve different mechanisms compared to other lung cancers (22).

## Limitations

This was a case report study, which was not highlight the long follow-up period, and lack of molecular diagnostic methods.

## Conclusion

In conclusion, this case illustrates the evolving imaging signs of lung IMA. Medical professionals should avoid diagnosing lung cancer as pneumonia, with the aim of enhancing the accuracy of early diagnosis and assisting in clinical evaluation. Additionally, it serves as a reference for patients to better understand this disease.

## Data Availability

The original contributions presented in this study are included in this article/supplementary material, further inquiries can be directed to the corresponding authors.
